# Supplementary material for: Adaptive rewiring shapes structure and stability in a three-guild herbivore-plant-pollinator network
Source: Commun Biol. 2024 Jan 16;7:103. doi: 10.1038/s42003-024-05784-8 (PMC10791747; doi:10.1038/s42003-024-05784-8)
Supplement: Supplementary file 2 — Supplemental Information [file 42003_2024_5784_MOESM2_ESM.pdf]

Supplementary Online Materials to

“Adaptive rewiring shapes structure and stability in a three-guild herbivore-plant-pollinator network”

Min Su, Qi Ma, Cang Hui

This Supplementary Information file contains:

Supplementary Notes 1-6 (i.e., Theory of network stability, Nestedness and Modularity, Definition of network complexity, Sensitivity tests, and z-test**)**

Supplementary Figures 1-15

Supplementary Tables 1-2

Supplementary Note 1. Theory of network stability

One frequently used method to calculate the stability of ecological networks as the resilience index, i.e., the absolute of maximum real part of all eigenvalues of the Jacobian matrix[1,2], i.e., $\left|\left(Re(\lambda)\right)_{max}\right|$. A system needs to be locally asymptotically stable before calculating its resilience (i.e., $Re(\lambda) < 0$), so $\left|\left(Re(\lambda)\right)_{max}\right|$ is equal to $-\left(Re(\lambda)\right)_{max}$. The Jacobian matrix of Eqn. 1 in the main text was defined as followings:

$$\mathcal{F} = \begin{bmatrix} \mathcal{F}^{MM} & \mathcal{F}^{MH} & \mathcal{F}^{MP} \\ \mathcal{F}^{HM} & \mathcal{F}^{HH} & \mathcal{F}^{HP} \\ \mathcal{F}^{PM} & \mathcal{F}^{PH} & \mathcal{F}^{PP} \end{bmatrix} = \begin{bmatrix} \partial\dot{M}/\partial M & \partial\dot{M}/\partial H & \partial\dot{M}/\partial P \\ \partial\dot{H}/\partial M & \partial\dot{H}/\partial H & \partial\dot{H}/\partial P \\ \partial\dot{P}/\partial M & \partial\dot{P}/\partial H & \partial\dot{P}/\partial P \end{bmatrix}.$$

where the components in the first row at equilibrium can be written as $\mathcal{F}^{MM} = \left\{\mathcal{F}_{ij}^{MM}\right\}_{S_M \times S_M}$, $\mathcal{F}_{ij}^{MM} = -\beta_{ij}^{M} M_i$; $\mathcal{F}^{MH} = 0$; $\mathcal{F}^{MP} = \left\{\mathcal{F}_{ij}^{MP}\right\}_{S_M \times S_P}$, and

$$\mathcal{F}_{ij}^{MP} = M_i \left(\frac{\gamma_{ij}}{1+h\sum_k \Theta_{ik}^{MP} P_k} - \frac{h\gamma_{ij}P_j}{\left(1+h\sum_k \Theta_{ik}^{MP} P_k\right)^2} - \Theta_{ij}^{MP} \sum_{k\neq j}^{S_P} \frac{h\gamma_{ik}P_k}{\left(1+h\sum_k \Theta_{ik}^{MP} P_k\right)^2} \right),$$

$$= M_i \left(\frac{\gamma_{ij}}{1+h\sum_k \Theta_{ik}^{MP} P_k} - \frac{h\Theta_{ij}^{MP}}{1+h\sum_k \Theta_{ik}^{MP} P_k} \sum_{k=1}^{S_P} \frac{\gamma_{ik}P_k}{1+h\sum_k \Theta_{ik}^{MP} P_k} \right),$$

$$= M_i \left(\frac{\gamma_{ij} + h\Theta_{ij}^{MP}\left(r_{M_i} - \sum_{k=1}^{S_M} \beta_{ik}^{M} M_k\right)}{1+h\sum_k \Theta_{ik}^{MP} P_k} \right).$$

Similarly, we can derive the components in the second row of Jacobian community matrix, i.e., $\mathcal{F}^{HM} = 0$; $\mathcal{F}^{HH} = \left\{\mathcal{F}_{ij}^{HH}\right\}_{S_H \times S_H}$, $\mathcal{F}_{ij}^{HH} = -\beta_{ij}^{H} H_i$; $\mathcal{F}^{HP} = \left\{\mathcal{F}_{ij}^{HP}\right\}_{S_H \times S_P}$, and $\mathcal{F}_{ij}^{HP} = H_i \left(\frac{\varepsilon\tau_{ij} + h\Theta_{ij}^{HP}\left(r_{H_i} - \sum_{k=1}^{S_H} \beta_{ik}^{H} H_k\right)}{1+h\sum_k \Theta_{ik}^{HP} P_k} \right)$.

The components in the third row of Jacobian community matrix, i.e., $\mathcal{F}^{PM} = \{\mathcal{F}_{ij}^{PM}\}_{S_P \times S_M}$, $\mathcal{F}_{ij}^{PM} = P_i\left(\frac{\gamma_{ij}+h\Theta_{ij}^{PM}\left(r_{P_i}-\sum_{j=1}^{S_P}\beta_{ij}^{P}P_j+\sum_{k=1}^{S_H}\frac{\tau_{ik}H_k}{1+h\sum_l \Theta_{kl}^{HP}P_l}\right)}{1+h\sum_k \Theta_{ki}^{MP}M_k}\right)$;

$\mathcal{F}^{PH} = \{\mathcal{F}_{ij}^{PH}\}_{S_P \times S_H}$, $\mathcal{F}_{ij}^{PH} = \frac{\tau_{ij}}{1+h\sum_k \Theta_{jk}^{HP}P_k}P_i$;

$\mathcal{F}^{PP} = \{\mathcal{F}_{ij}^{PP}\}_{S_P \times S_P}$, $\mathcal{F}_{ij}^{PP} = P_i\left(-\beta_{ij}^{P} - \sum_{k=1}^{S_H}\frac{h\tau_{ik}\Theta_{kj}^{HP}H_k}{\left(1+h\sum_l \Theta_{kl}^{HP}P_l\right)^2}\right)$

Therefore, we can calculate the absolute of maximum real part of all eigenvalues of the Jacobian matrix ($-\left(Re(\lambda)\right)_{max}$) at the assembly stable communities, i.e., calculated by species interaction matrix and species biomass at the end of 10^5 replicates of interactions switching[3].

Supplementary Note 2. Nestedness and Modularity

The structures of modeled ecological networks were measured by nestedness based on the overlap and decreasing fill (NODF)[4,5] and modularity using Newman (2006) algorithm[6]. The nestedness is measured as follows:

$$NODF = \frac{\sum N_{paired}}{\left[\frac{S_A(S_A-1)}{2}\right] + \left[\frac{S_P(S_P-1)}{2}\right]},$$

where S_A (i.e., S_M or S_H) and S_P represents species number of animal (pollinator or herbivore) and plant. The degree of paired nestedness (N_{paired}) is determined by the decreasing filling and paired overlap of the adjacency matrix Θ^{MP} (Θ^{HP}).

For pollination or herbivory sub-networks, the modularity of the adjacency matrix $\Theta^{MP} = \{\theta_{ij}\}_{S_M \times S_P}$ or $\Theta^{HP} = \{\theta_{ij}\}_{S_H \times S_P}$ is calculated by seeking a partition of the network that maximizes the modularity quality function[3,6]:

$$Q = \frac{1}{2L} \sum_{i \in A, j \in P} \left(\theta_{ij} - \frac{k_i k_j}{2L} \right) \delta(c_i, c_j),$$

where k_i, k_j are the degree of animal i and plant j, $2L$ is the total number of links in sub-networks, c_i is the module that node i belongs to a certain partition, the δ function $\delta(c_i, c_j)$ is 1 if $c_i = c_j$ and 0 otherwise. For each sub-network in the 3-guild adapting network, we used the leading eigenvector algorithm to obtain the modularity value[3,6]. We used the open-source Matlab package BiMat for calculating nestedness and modularity of 3-guild networks[7].

Supplementary Note 3. Relative Nestedness and Modularity

In order to assess whether the changes in observed nestedness and modularity are related to network complexity (i.e., changes in species richness and network connectance), we calculated the relative nestedness and relative modularity[8] as follows:

$$N^* = \frac{N - \overline{N}_r}{\overline{N}_r}$$

$$Q^* = \frac{Q - \bar{Q}_r}{\bar{Q}_r}$$

where N^* and Q^* are the relative nestedness and modularity, and N, Q are the observed nestedness and modularity, $\overline{N}_r$ and $\bar{Q}_r$ are the average nestedness and modularity of 1000 replicates of the null model. Here, the null model assumes the connectance of mutualistic or antagonistic sub-network is respected, and any link between each pair of plant species i and animal (pollinator or herbivore) j occurs with the same probability ($P_{ij} = L/(S_P \times S_M)$ or $P_{ij} = L/(S_P \times S_H)$) equal to connectance of the observed sub-network[7]. Then, we can obtain the relative architectures of mutualistic and antagonistic sub-networks (N_l^*, Q_l^*; $l = ant$ or mut), and these measures can show how nested and modular are the network when compared with the mean expected nestedness and modularity under a given null model with the same complexity[1,8,9].

Supplementary Note 4. Definition of network complexity with asymmetrical sub-networks

The complexity of two sub-networks may play an important role in determine the 3-guild network structure[1]. To understand how the different structural patterns of mutualistic and antagonistic sub-networks affect the properties of 3-guild networks, we held plant richness constant ($S_P = 30$) and performed two types of simulations varying animal species richness and sub-networks' connectance as followings:

(1) We define four cases for the structure of the antagonistic sub-network, combining different cases of species richness and connectance: $\{S_H,\ C_{ant}\}$= [{15, 0.1}, {45, 0.1}, {15, 0.25}, {45, 0.25}].

(2) We define four cases for the structure of the mutualistic sub-network, combining different cases of species richness and connectance: $\{S_M,\ C_{mut}\}$= [{15, 0.1}, {45, 0.1}, {15, 0.25}, {45, 0.25}].

The species richness of antagonistic and mutualistic sub-networks is defined as follows: $S_{ant} = S_H + S_P,\ S_{mut} = S_M + S_P$. For simplicity, we define the complexity of a sub-network as the product of its size and connectance[1]: the antagonistic complexity is $S_{ant} \times C_{ant}$, and the mutualistic complexity is $S_{mut} \times C_{mut}$.

Supplementary Note 5. Sensitivity tests of main parameters in the model.

To test the robustness of our main findings, we used a Latin hypercube sampling (LHS) to explore a region of the parameter space for sensitivity tests[10]. The Latin hypercube is a sampling method that aims to produce a random distribution of points in the high dimensional parameter space. It provides an unbiased assessment of the average model output, with the advantage that it requires fewer samples than simple random sampling to achieve the same level of test robustness. From a region of the 6-dimensional parameter space, i.e., for S_x $(x = P, H, M)$, $C_l(l = mut, ant)$, $r_{x_i}(x = P, H, M)$, h, ε and σ defined by $\pm 30\%$ of the baseline parameter values (used in the main text), 10 sets of parameter samples were drawn to explore the effects of composition of multiple interaction types on the resilience of 3-guild networks (Supplementary Table 2).

In addition, to obtain a robust view of network resilience therein, we tested effects of two key parameters (S_x, C_l) in a wide range. We performed two types of simulations varying species richness and sub-networks' connectance as followings:

(1) We held connectance constant $(C_l = 0.15)$ and performed three cases of species richness $S_x = \{15, 30, 45\}$, combining $\Omega_c = \{0.01, 0.1\}$ and $\Omega_m = \{0.1, 0.35\}$;

(2) We held species richness constant $(S_x = 30)$ and performed three cases of sub-network's connectance $C_l = \{0.1, 0.15, 0.2\}$, combining $\Omega_c = \{0.01, 0.1\}$ and $\Omega_m = \{0.1, 0.35\}$.

Through randomly selecting a set of parameter combinations, sensitivity tests showed that the main results are qualitatively robust against the parameters in model (Supplementary Fig. 3a). Meanwhile, compared analysis of resilience under two levels of mutualistic strength ($\Omega_m = 0.1, 0.35$) also showed that enhancing mutualism could stabilize the symmetric 3-guild network at low competition level ($\Omega_c = 0.01$), regardless varying species richness or sub-network's connectance (Supplementary Fig. 3b, d). When holding a high competition ($\Omega_c = 0.1$), enhancing mutualism could destabilize the 3-guild network as suggested in Fig. 2 (Supplementary Fig. 3c, e). Overall, the role of interaction strengths in driving network resilience appeared robust under other model parameters.

Supplementary Note 6. z-test

Using z-test approach to test whether adaptive networks will be significantly more nested or more compartmentalized than that of null model. The 1000 replicates of null model are generated as a 'population', then we can obtain the mean of population (μ_0) and the standard deviation (σ_0). Taking the nestedness (or modularity) of adaptive networks from n replicates as a sample, then the mean of sample denoted as $\overline{X}$, and size of sample is n (here is 60). Hypothesis H_0: $\mu = \mu_0$, H_1: $\mu > \mu_0$. Under one tailed-z-test and 95% confidence interval, if the score of statistic $Z = \frac{\overline{X}-\mu_0}{\sigma_0/\sqrt{n}}$ is above 1.645 (z-score>1.645), then we reject hypothesis H_0, and can obtain that adaptive networks will be significantly more nested or more compartmentalized than that of null model.

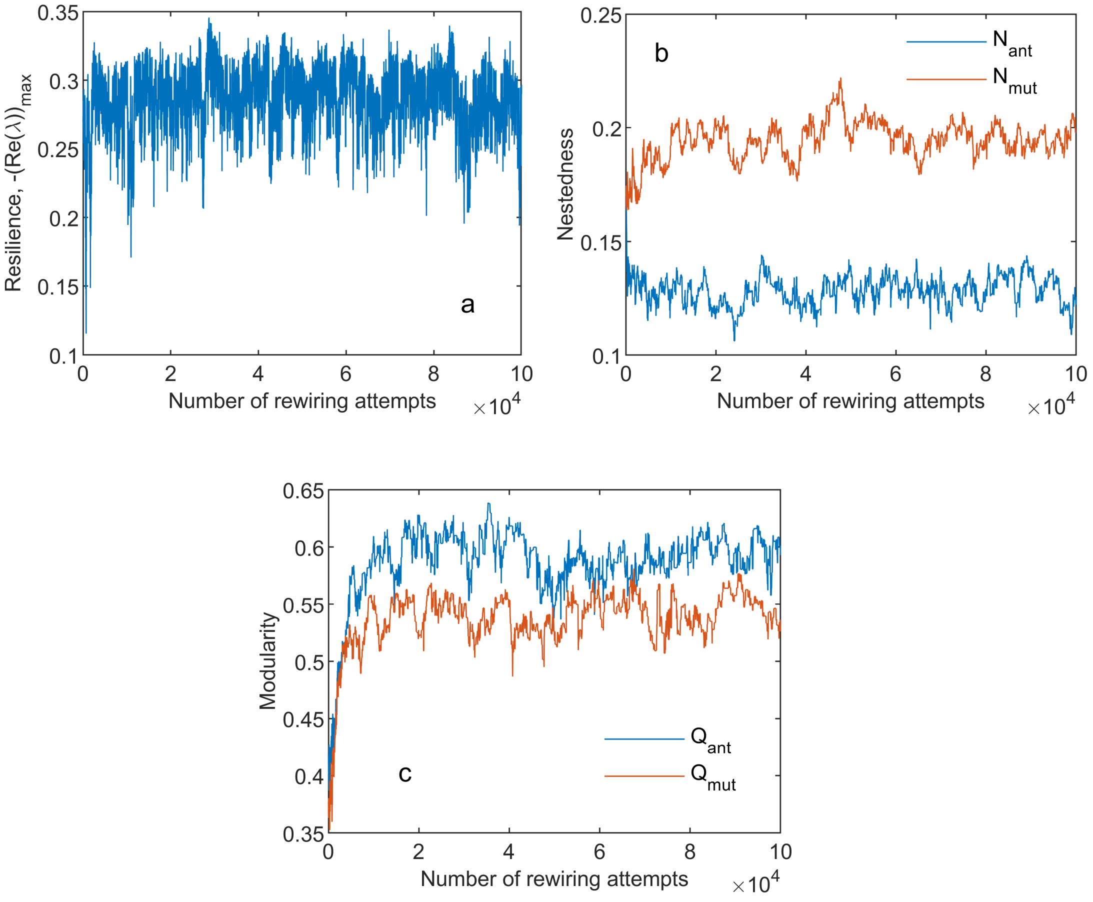

Supplementary Fig 1. Equilibrium dynamics of network resilience (a), sub-networks' nestedness (b) and modularity (c) in the adaptive network, with the horizontal axis representing the number of rewiring attempts. Parameters are the same with Fig. 1b.

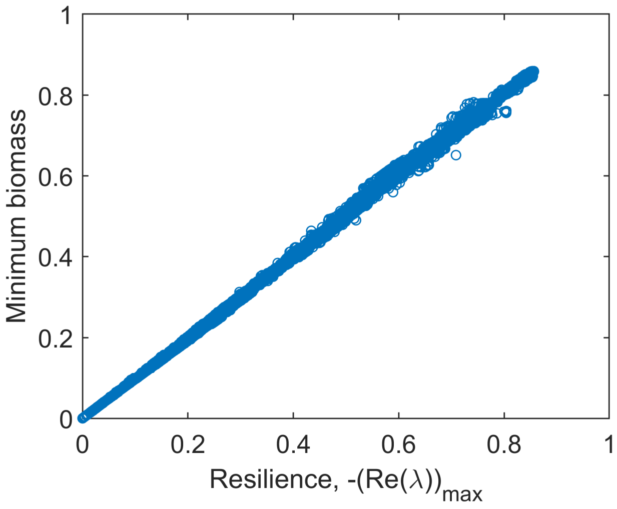

Supplementary Fig 2. Relationship between resilience and minimum biomass of species in 3-guild networks with 9 interaction composition of $\{\Omega_c, \Omega_m, \Omega_p\}$ during the final 10^4 rewiring attempts (9×10^4 data points in total). Parameter combinations are $\Omega_c = 0.1$, $\Omega_m = 0.1, 0.2, 0.3$, $\Omega_p = 0.05, 0.15, 0.25$.

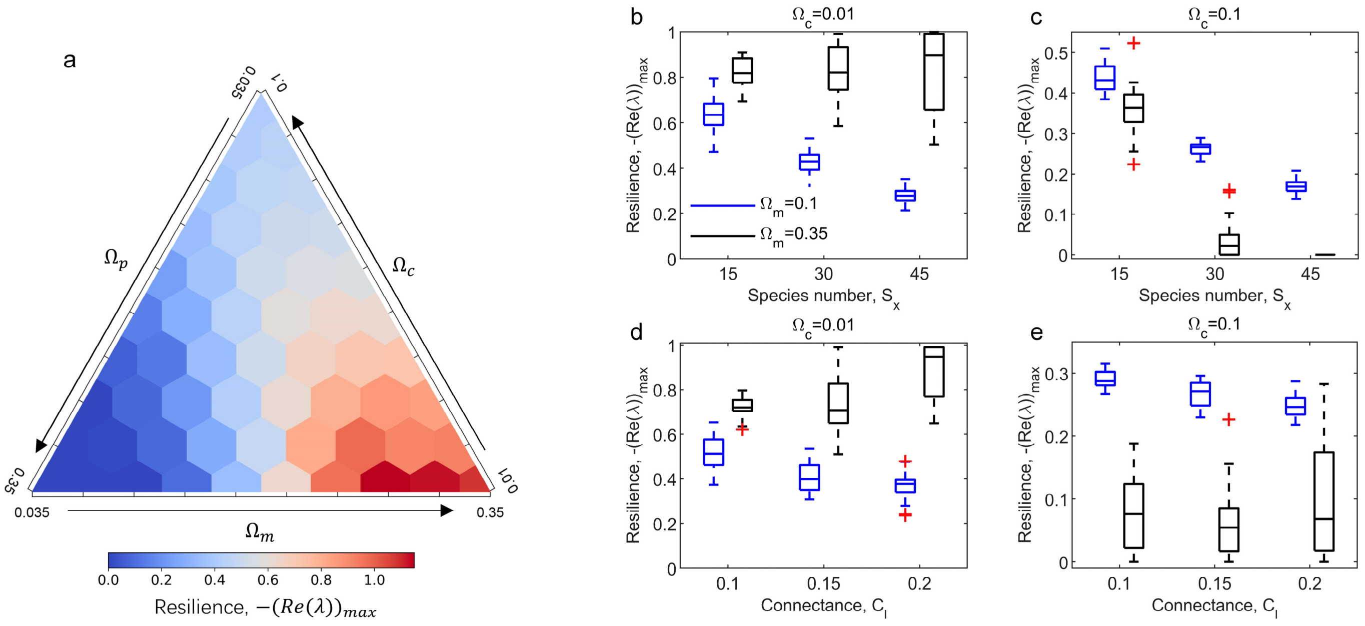

Supplementary Fig 3. The robust response of network resilience to interaction strengths. (a) Sensitivity tests of network resilience responding to interaction strengths under parameter set 1 of Supplementary Table 2. (b, c) Resilience responds to mutualistic strength ($\Omega_m = \{0.1, 0.35\}$) under three levels of species richness ($S_x = \{15, 30, 45\}$) when holding strength of competition at 0.01 and 0.1. (d, e) Resilience responds to mutualistic strength ($\Omega_m = \{0.1, 0.35\}$) under three levels of sub-network's connectance ($C_l = \{0.1, 0.15, 0.2\}$) when holding strength of competition at 0.01 and 0.1. Blue and black lines represent $\Omega_m = 0.1, 0.35$, respectively. $\Omega_p = 0.15$ and other parameters in (b-e) are the same with Table 1.

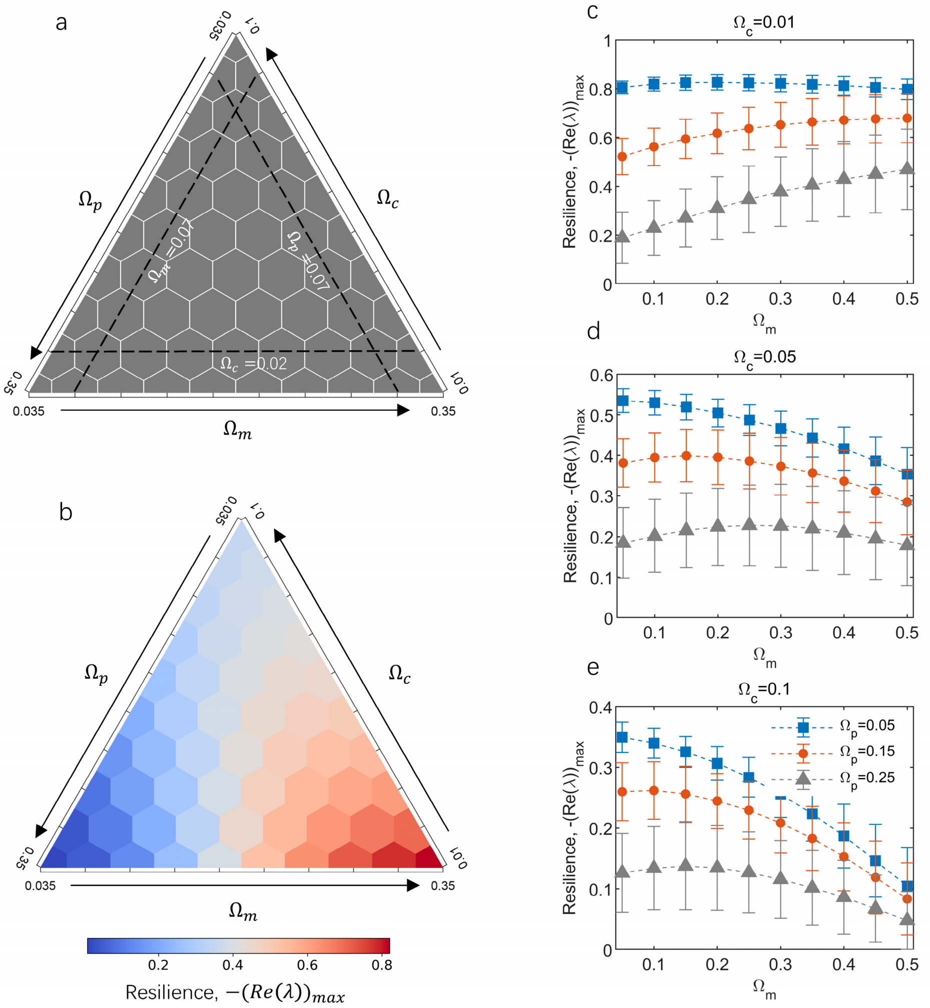

Supplementary Fig 4. The response of network resilience of random interaction networks ($-(Re(\lambda))_{max}$) to combinations of interaction strengths. (a) A schematic guide to read the ternary plot. The three dashed lines represent the strength of a specified interaction type held constant. The centre of each hexagon represents an examined combination of interaction strength (55 in total); (b) The mean resilience for each combination, calculated from 60 replicates; (c-e) Resilience responds to increasing mutualistic strength when holding competition and antagonism constant. Data in (c-e) are obtained from 60 simulation replicates and presented as mean values±SD. Parameters are the same with Fig. 2 in the main text.

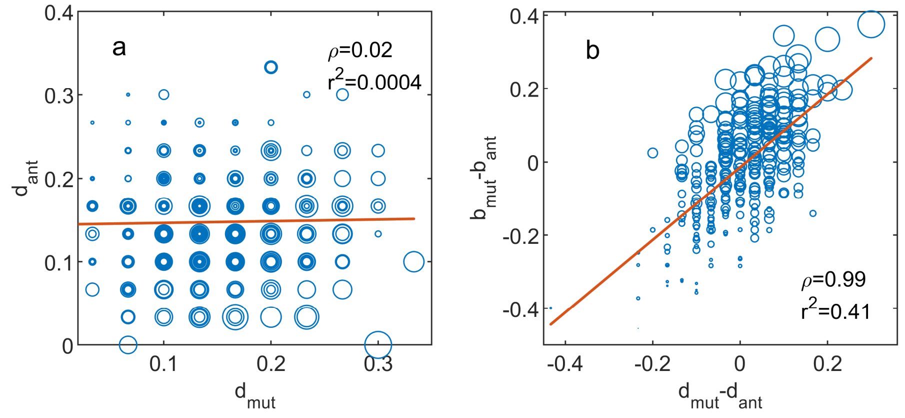

Supplementary Fig 5. The relationship of plants' degree centrality in mutualistic and antagonistic network, and degree centrality-energy budget (scatter diagram of $d_{mut} - d_{ant}$ and $b_{mut} - b_{ant}$) under random networks with arbitrary partnerships. Red lines represent least-square fits under respective scenarios. Circle sizes in Supplementary Fig. 5a and 5b are proportional to species biomass. Other parameters are the same with Fig. 3a and Fig. 3d.

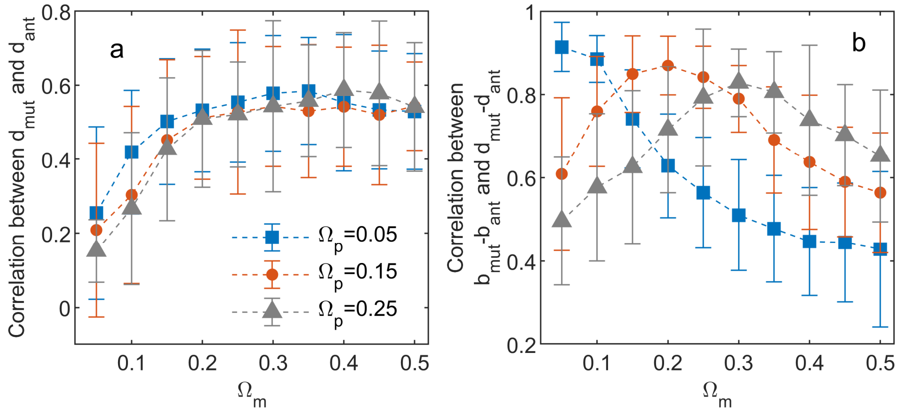

Supplementary Fig 6. The response of plants' degree centrality in mutualistic and antagonistic network, and degree centrality-energy budget correlations to mutualistic interaction strengths. Competitive strength $\Omega_c = 0.01$, and other parameters are the same with Fig. 3b and Fig. 3e in the main text.

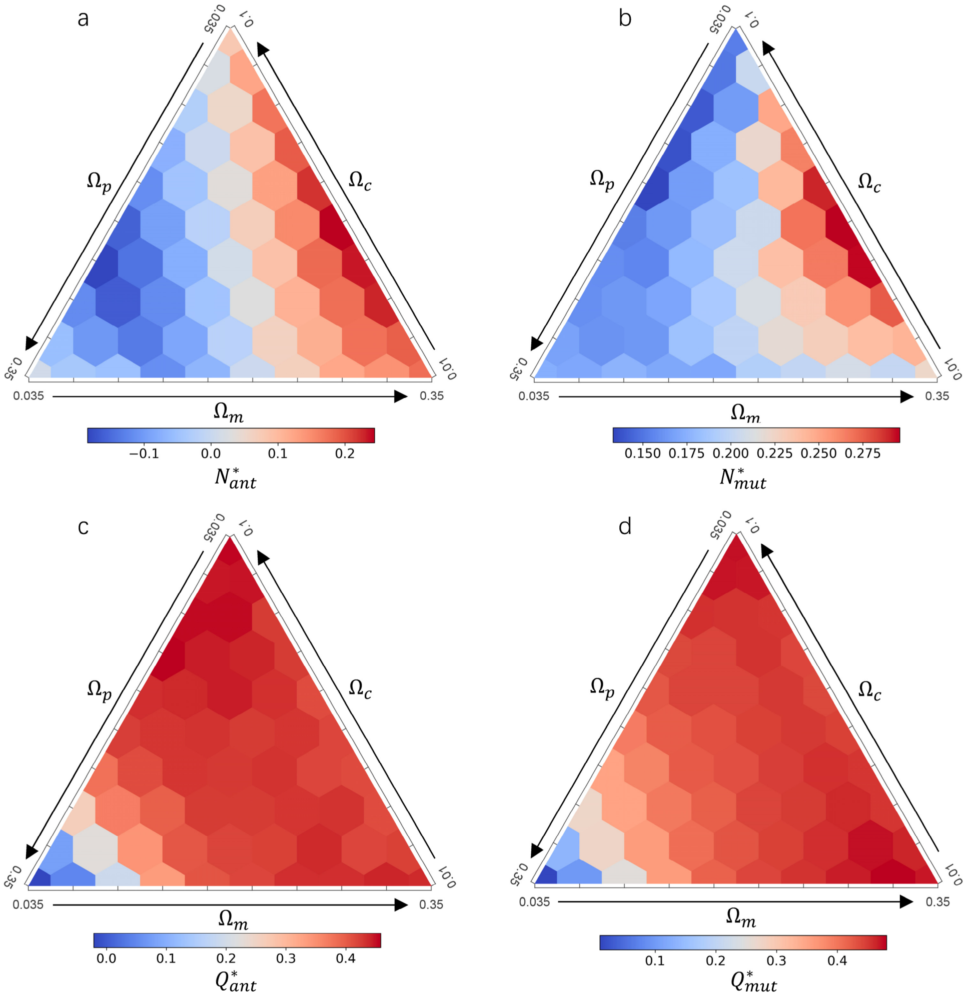

Supplementary Fig 7. The response of relative nestedness and relative modularity of the antagonistic sub-network and the mutualistic sub-network to the combination of interaction strengths. (a, b) the mean of the relative antagonistic nestedness (N_{ant}^{*}) and relative mutualistic nestedness (N_{mut}^{*}) for each combination of interaction strengths, calculated from 60 replicates. (c, d) the mean of the relative antagonistic modularity (Q_{ant}^{*}) and relative mutualistic modularity (Q_{mut}^{*}) for each combination of interaction strengths. Results are similar to the response of nestedness and modularity to combinations of interaction strengths in Fig. 4.

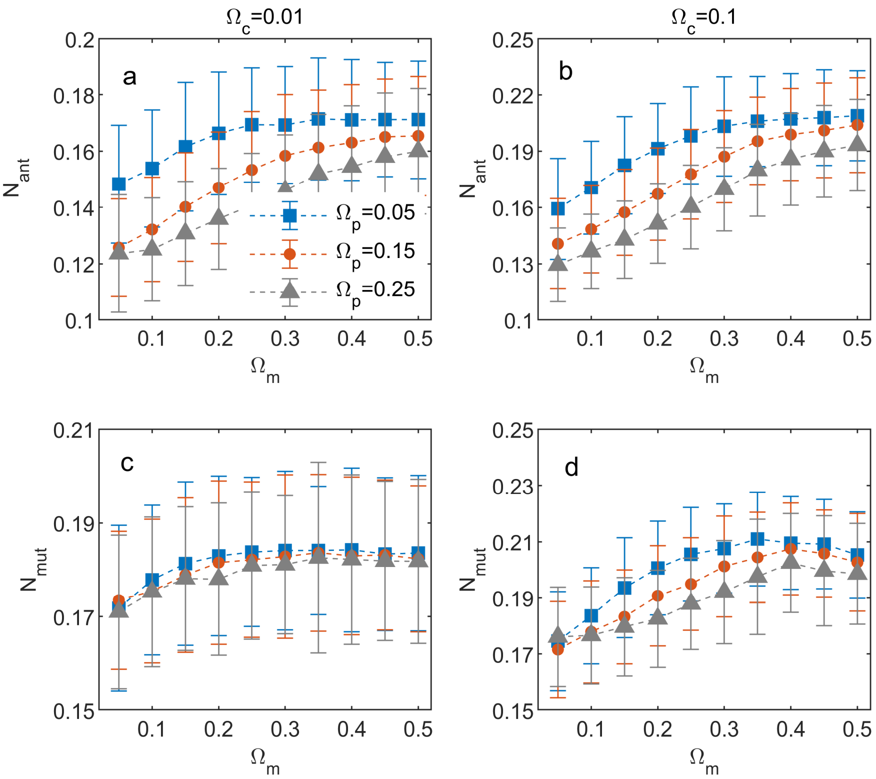

Supplementary Fig 8. The response of nestedness of mutualistic and antagonistic networks to the strength of mutualistic interaction. (a, c) effects of mutualistic strength on sub-networks' nestedness at $\Omega_c = 0.01$; (b, d) effects of mutualistic strength on sub-networks' nestedness when holding competitive strength at 0.1. Data are obtained from 60 simulation replicates and presented as mean values±SD. Other parameters are the same with Fig. 4 in the main text.

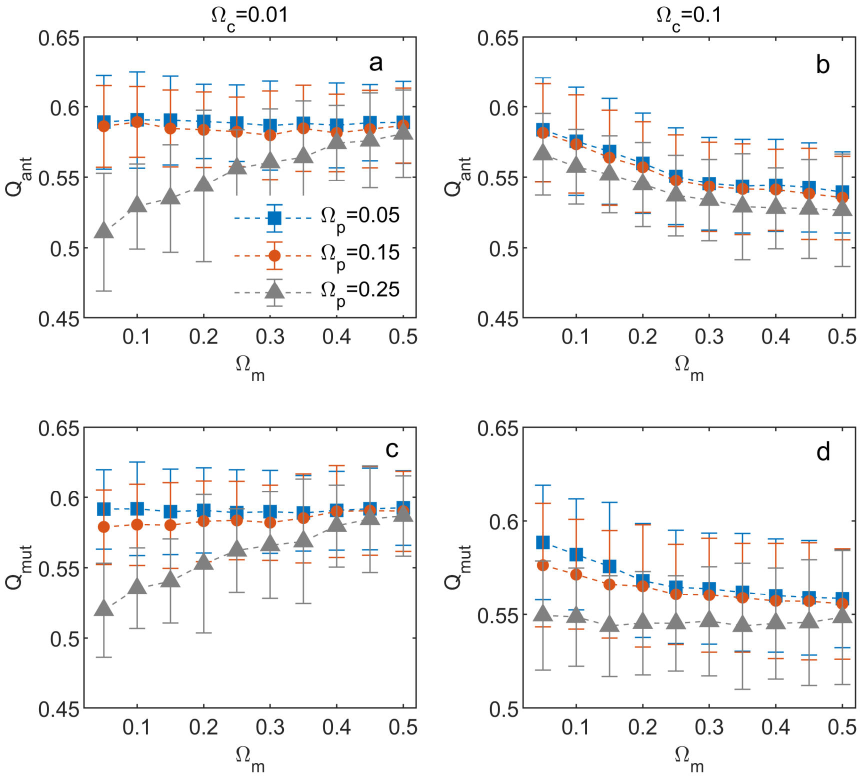

Supplementary Fig 9. The response of modularity of mutualistic and antagonistic networks to the strength of mutualistic interaction. (a, c) effects of mutualistic strength on sub-networks' modularity at $\Omega_c = 0.01$; (b, d) effects of mutualistic strength on sub-networks' modularity when holding competitive strength at 0.1. Data are obtained from 60 simulation replicates and presented as mean values±SD. Other parameters are the same with Fig. 4 in the main text.

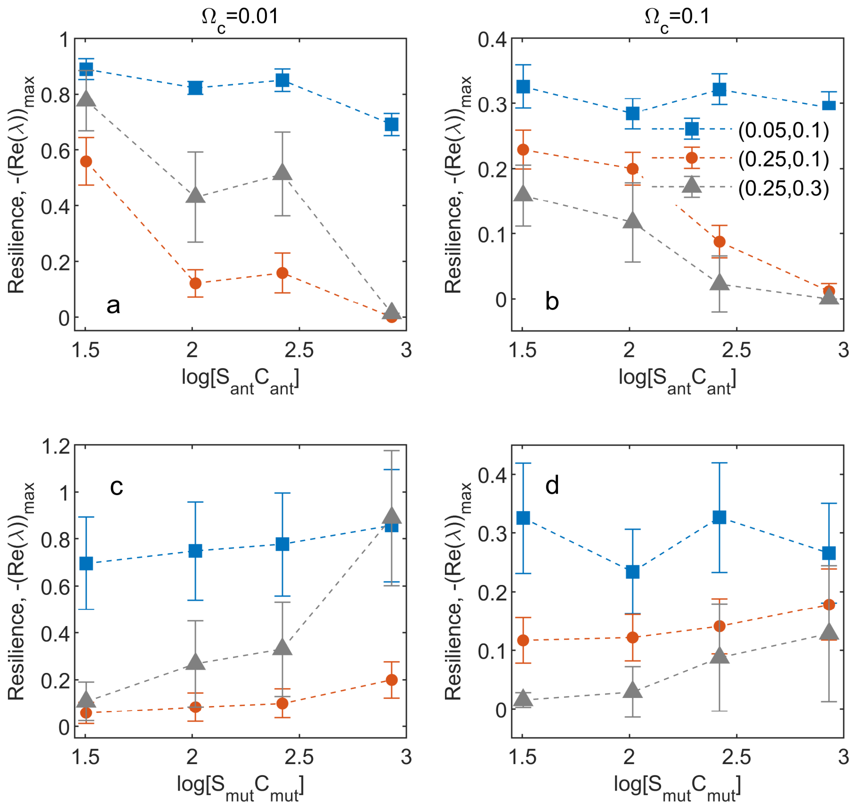

Supplementary Fig 10. The response of stability (measured as resilience, $-\left(Re(\lambda)\right)_{max}$) to interaction strengths with various sub-networks' complexities. (a, b) Resilience responds to interaction strengths and antagonistic sub-network's complexity when holding strength of competition at 0.01 and 0.1. (c, d) Resilience responds to interaction strengths and mutualistic sub-network's complexity when holding strength of competition at 0.01 and 0.1. Blue, red and grey lines represent $(\Omega_p, \Omega_m) = (0.05, 0.1)$, $(0.25, 0.1)$, and $(0.25, 0.3)$, respectively. Data are obtained from 60 simulation replicates and presented as mean values±SD.

Note: Demonstrations of results for the competitive strength at 0.01 in Supplementary Figs. 11-15, and parallel results when holding competitive strength at 0.1.

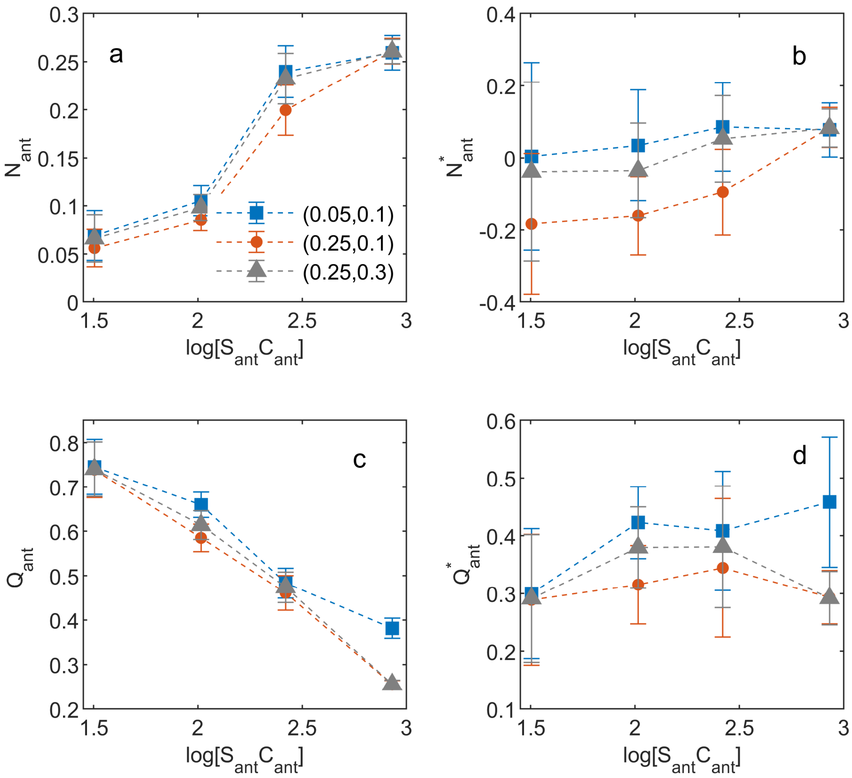

Supplementary Fig 11. The response of antagonistic sub-network structures to interaction strengths with various antagonistic complexities. (a, b) Nestedness (N_{ant}) and relative nestedness (N_{ant}^{*}) of antagonistic sub-network respond to interaction strengths and antagonistic complexity. (c, d) Modularity (Q_{ant}) and relative modularity (Q_{ant}^{*}) of antagonistic sub-network respond to interaction strengths and antagonistic complexity. Blue, red and grey lines represent $(\Omega_p, \Omega_m) = (0.05, 0.1)$, $(0.25, 0.1)$, and $(0.25, 0.3)$, respectively. Data are obtained from 60 simulation replicates and presented as mean values±SD.

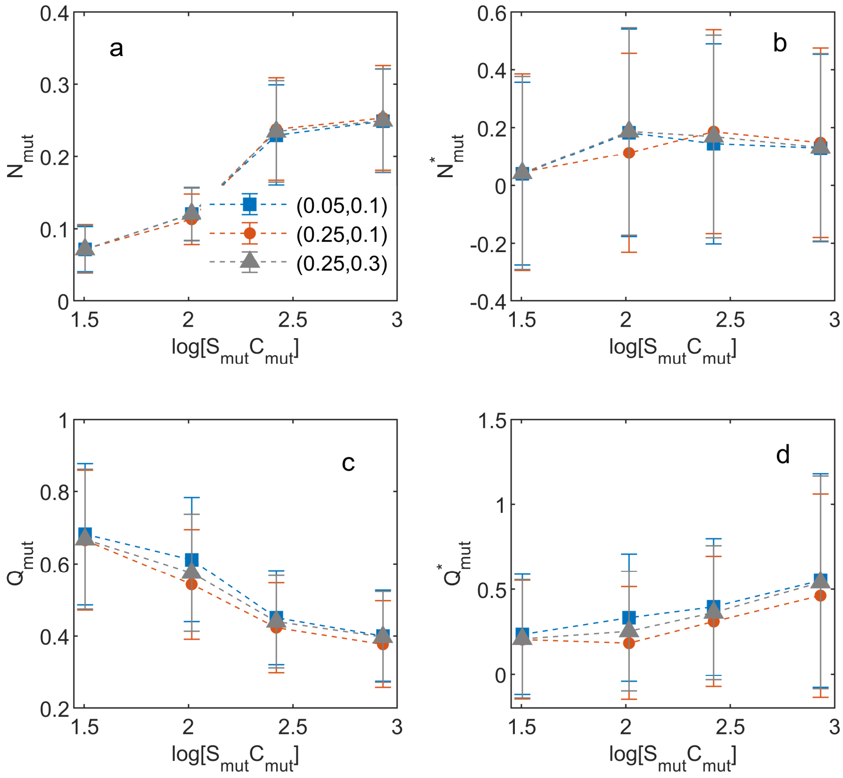

Supplementary Fig 12. The response of mutualistic sub-network structures to interaction strengths with various mutualistic complexities. (a, b) Nestedness (N_{mut}) and relative nestedness (N^*_{mut}) of mutualistic sub-network respond to interaction strengths and mutualistic complexity. (c, d) Modularity (Q_{mut}) and relative modularity (Q^*_{mut}) of mutualistic sub-network respond to interaction strengths and mutualistic complexity. Blue, red and grey lines represent $(\Omega_p, \Omega_m) = (0.05, 0.1)$, $(0.25, 0.1)$, and $(0.25, 0.3)$, respectively. Data are obtained from 60 simulation replicates and presented as mean values±SD.

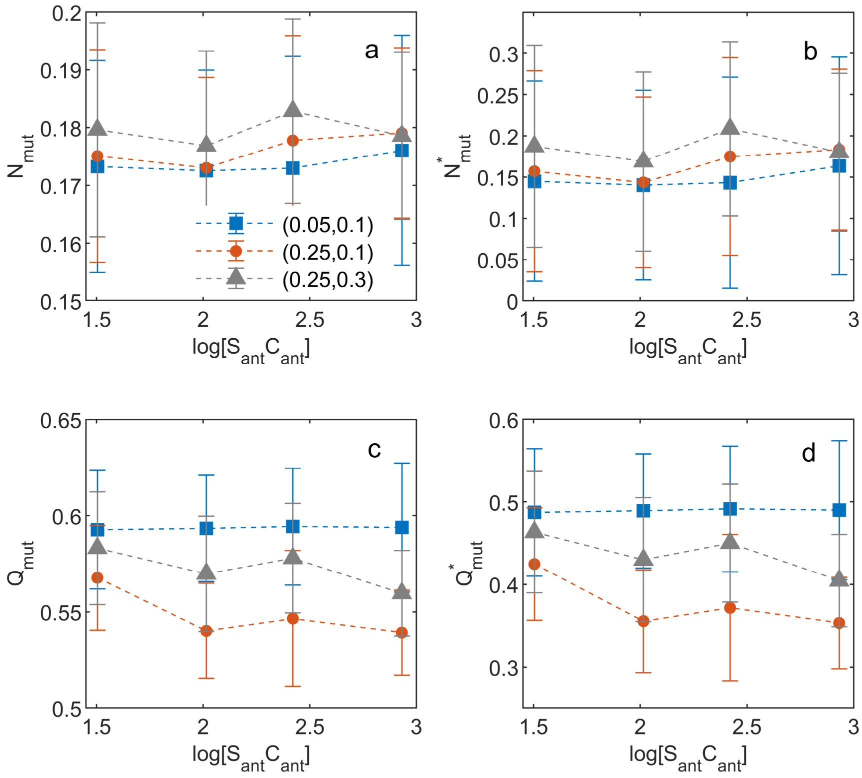

Supplementary Fig 13. The response of mutualistic sub-network structures to interaction strengths with various antagonistic complexities. (a, b) Nestedness (N_{mut}) and relative nestedness (N^*_{mut}) respond to interaction strengths and antagonistic complexity. (c, d) Modularity (Q_{mut}) and relative modularity (Q^*_{mut}) respond to interaction strengths and antagonistic complexity. Blue, red and grey lines represent $(\Omega_p, \Omega_m) = (0.05, 0.1)$, $(0.25, 0.1)$, and $(0.25, 0.3)$, respectively. Data are obtained from 60 simulation replicates and presented as mean values$\pm$SD.

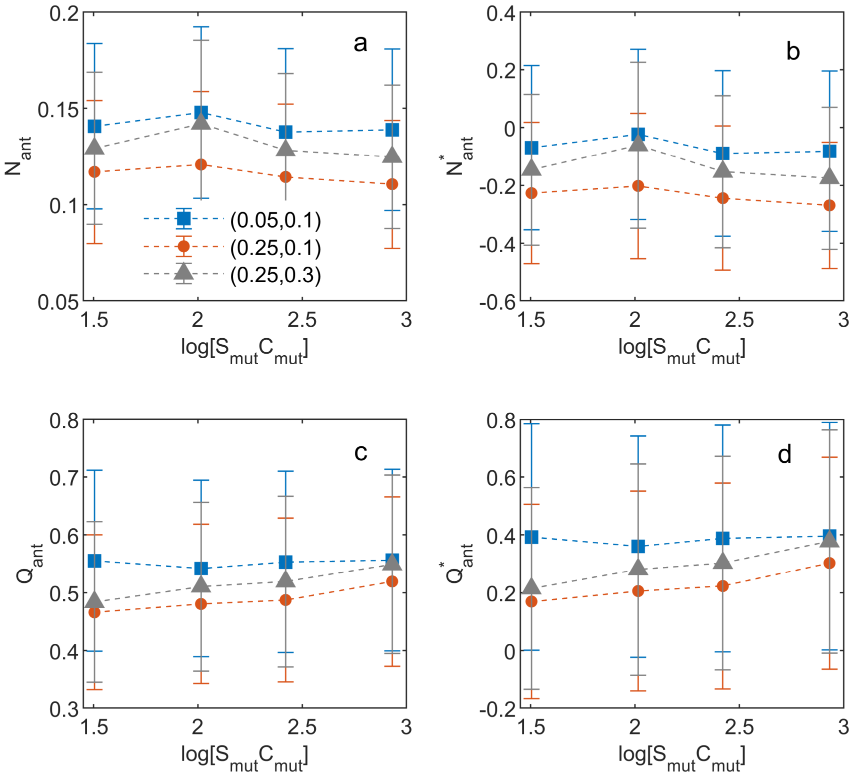

Supplementary Fig 14. The response of antagonistic sub-network structures to interaction strengths with various mutualistic complexities. (a, b) Nestedness (N_{ant}) and relative nestedness (N^*_{ant}) respond to interaction strengths and mutualistic complexity. (c, d) Modularity (Q_{ant}) and relative modularity (Q^*_{ant}) respond to interaction strengths and mutualistic complexity. Blue, red and grey lines represent $(\Omega_p, \Omega_m) = (0.05, 0.1)$, $(0.25, 0.1)$, and $(0.25, 0.3)$, respectively. Data are obtained from 60 simulation replicates and presented as mean values$\pm$SD.

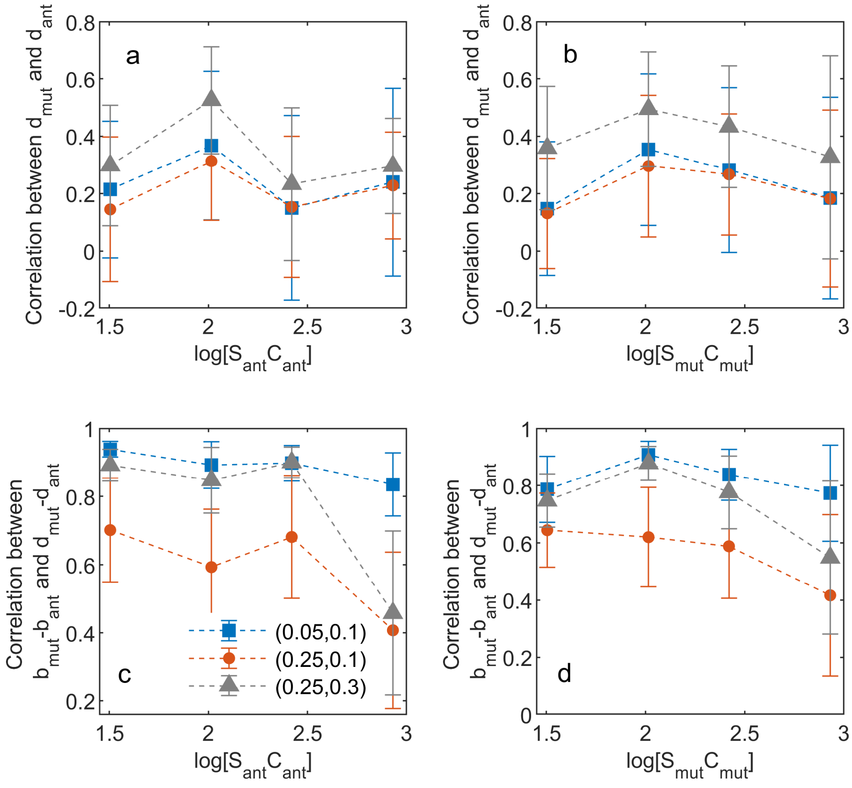

Supplementary Fig 15. The response of correlations to interaction strengths with various sub-networks' complexities. (a, b) Correlations between d_{mut} and d_{ant} respond to interaction strengths and sub-networks' complexities. (c, d) Correlations between $b_{mut} - b_{ant}$ and $d_{mut} - d_{ant}$ respond to interaction strengths and sub-networks' complexities. Blue, red and grey lines represent $(\Omega_p, \Omega_m) = (0.05, 0.1)$, $(0.25, 0.1)$, and $(0.25, 0.3)$, respectively. Data are obtained from 60 simulation replicates and presented as mean values±SD.

Supplementary Table 1. Number and frequency of species with low biomass during the final 10^4 rewiring attempts.

Threshold of minimum biomass	Number of species	Frequency of species
$< 10^{-4}$	0	0
$< 10^{-3}$	3	3.33×10^{-5}
$< 10^{-2}$	12	1.33×10^{-4}

Supplementary Table 2. Parameter sets from Latin hypercube sampling (LHS) for model sensitivity tests.

Parameters	S_x $(x = P, M, H)$	C_l $(l = mut, ant)$	r_{x_i} $(x = P, H, M)$	h	ε	σ
baseline	30	0.15	1	0.1	0.8	0.1
set 1	36	0.171094125	1.182001563	0.113880237	0.729512976	0.094315364
set 2	21	0.107233724	1.001855376	0.095434075	0.620715168	0.119786687
set 3	35	0.119649544	0.986463883	0.080189593	1.001053893	0.100639873
set 4	25	0.164379602	0.743849525	0.088211221	0.600149252	0.070175974
set 5	29	0.127251728	0.818661537	0.087206989	0.948721636	0.076865563
set 6	27	0.181966194	1.152164842	0.119878639	0.805392442	0.117299405
set 7	31	0.187702695	1.209580378	0.100469113	0.935651855	0.107899559
set 8	37	0.176723514	0.846918451	0.124846169	0.874107885	0.126495325
set 9	24	0.147896011	1.119815486	0.070701164	0.714537504	0.096286309
set 10	21	0.107233724	1.001855373	0.113880237	0.620744976	0.093353641

Supplementary References

1. Sauve, A. M. C., Fontaine, C. & Thébault, E. Structure-stability relationships in networks combining mutualistic and antagonistic interactions. Oikos 123, 378-384 (2014).
2. May, R. M. Will a large complex system be stable? Nature 238, 413-414 (1972).
3. Cai, W., Snyder, J., Hastings, A. & D'Souza, R. M. Mutualistic networks emerging from adaptive niche-based interactions. Nat. Commun. 11, 5470 (2020).
4. Bascompte, J., Jordano, P., Melián, C. J. & Olesen, J. M. The nested assembly of plant-animal mutualistic networks. Proc. Natl Acad. Sci. USA 100, 9383-9387 (2003).
5. Almeida-Neto, M., Guimarães, P., Guimarães Jr, P. R., Loyola, R. D. & Ulrich, W. A consistent metric for nestedness analysis in ecological systems: reconciling concept and measurement. Oikos 117, 1227-1239 (2008).
6. Newman, M. E. J. Modularity and community structure in networks. Proc. Natl. Acad. Sci. USA 103, 8577-8582 (2006).
7. Flores, C. O., Poisot, T., Valverde, S. & Weitz, J. S. BiMat: a MATLAB package to facilitate the analysis of bipartite networks. Methods Ecol. Evol. 7, 127-132 (2016).
8. Thébault, E. & Fontaine, C. Stability of ecological communities and the architecture of mutualistic and trophic networks. Science 329, 853-856 (2010).
9. Zhang, F., Hui, C. & Terblanche, J. S. An interaction switch predicts the nested architecture of mutualistic networks. Ecol. Lett. 14, 797-803 (2011).
10. Marino, S., Hogue, I. B., Ray, C. J., & Kirschner, D. E. A methodology for performing global uncertainty and sensitivity analysis in systems biology. J. Theor. Biol. 254, 178-196 (2008).
